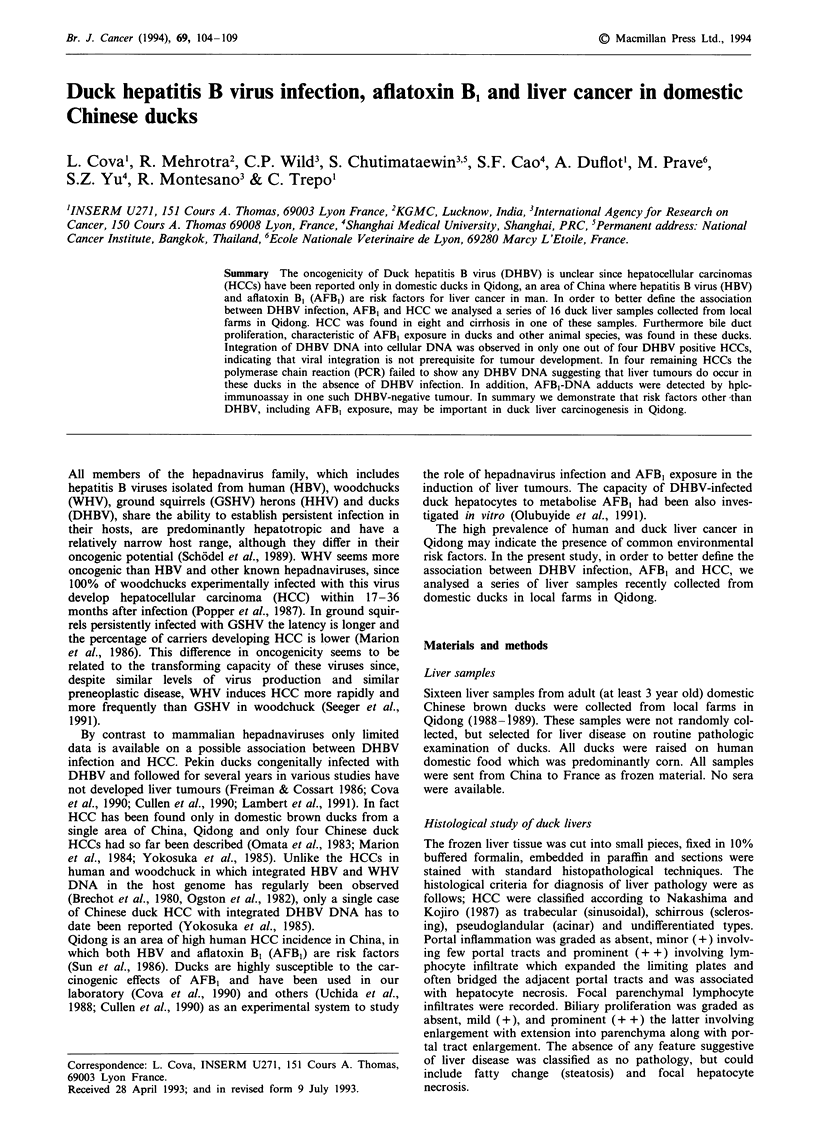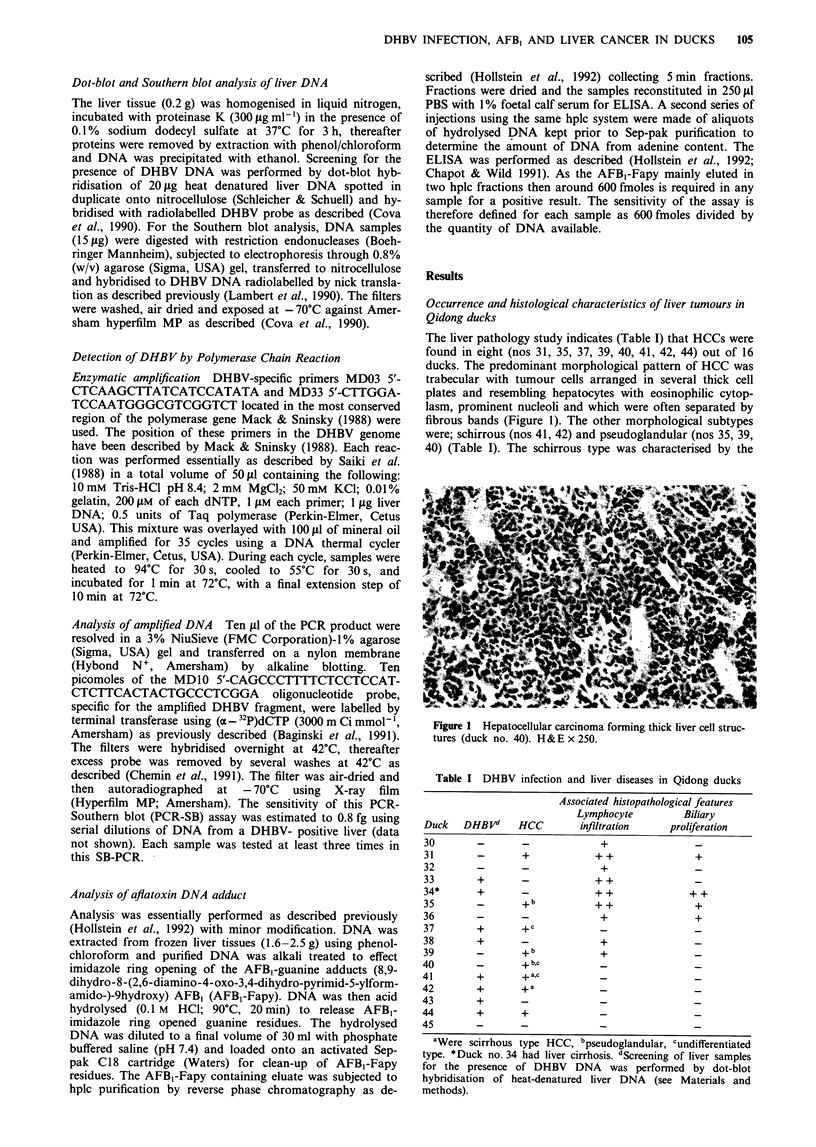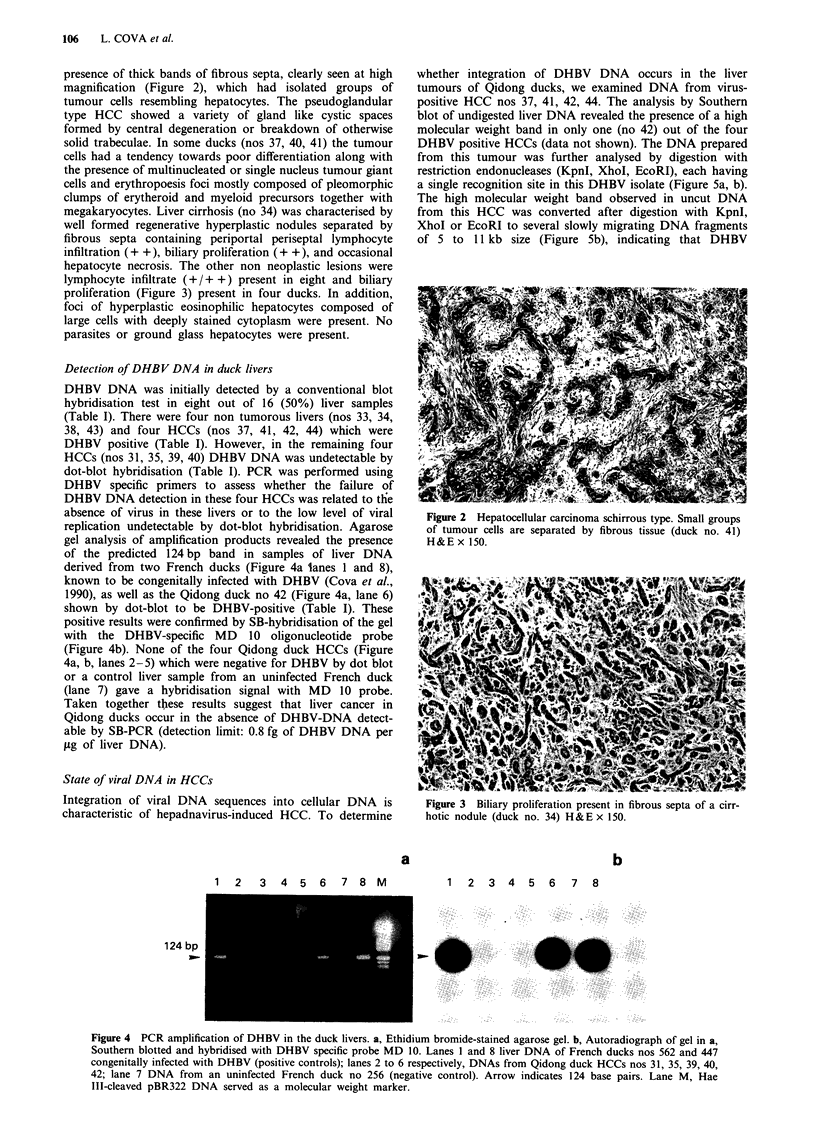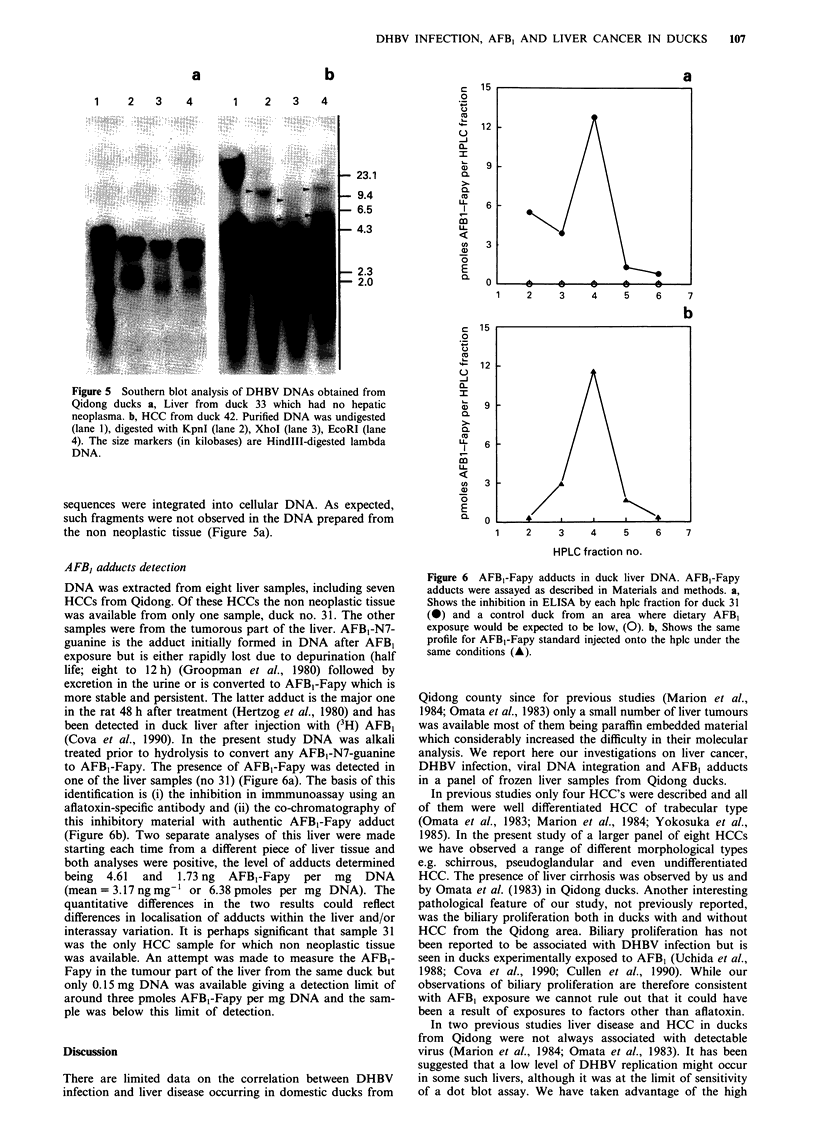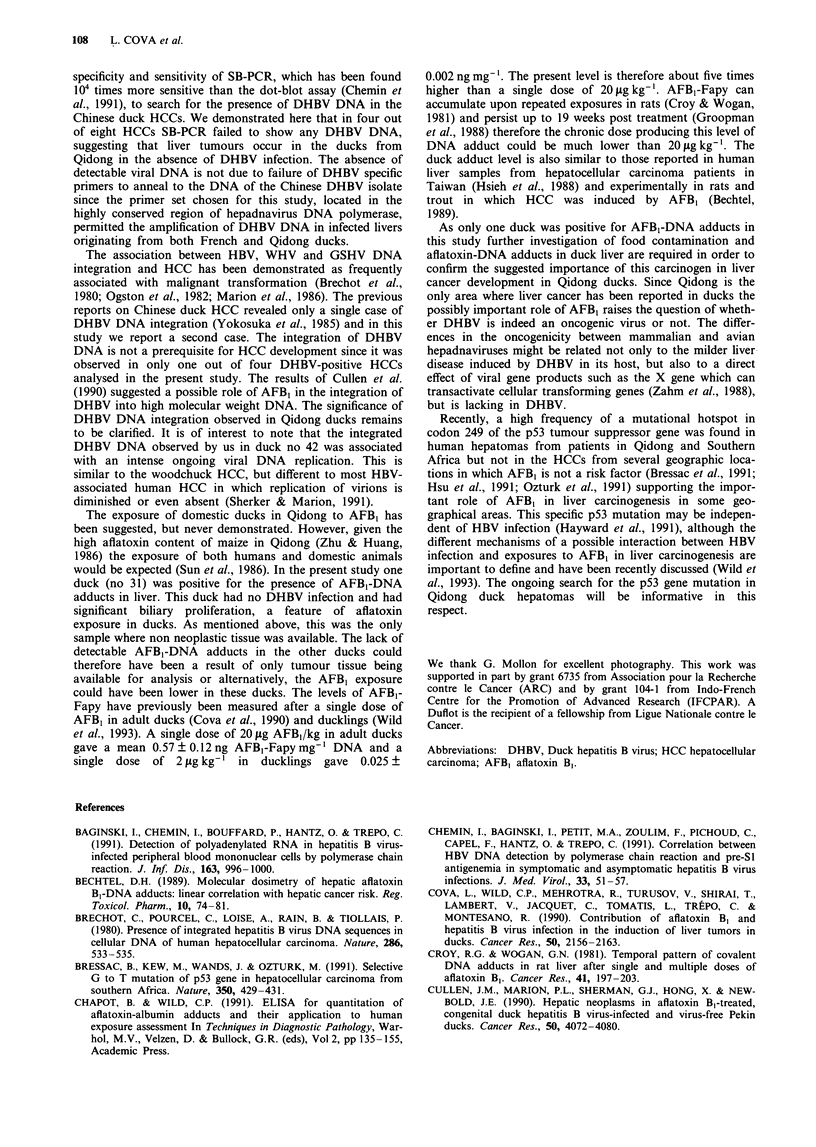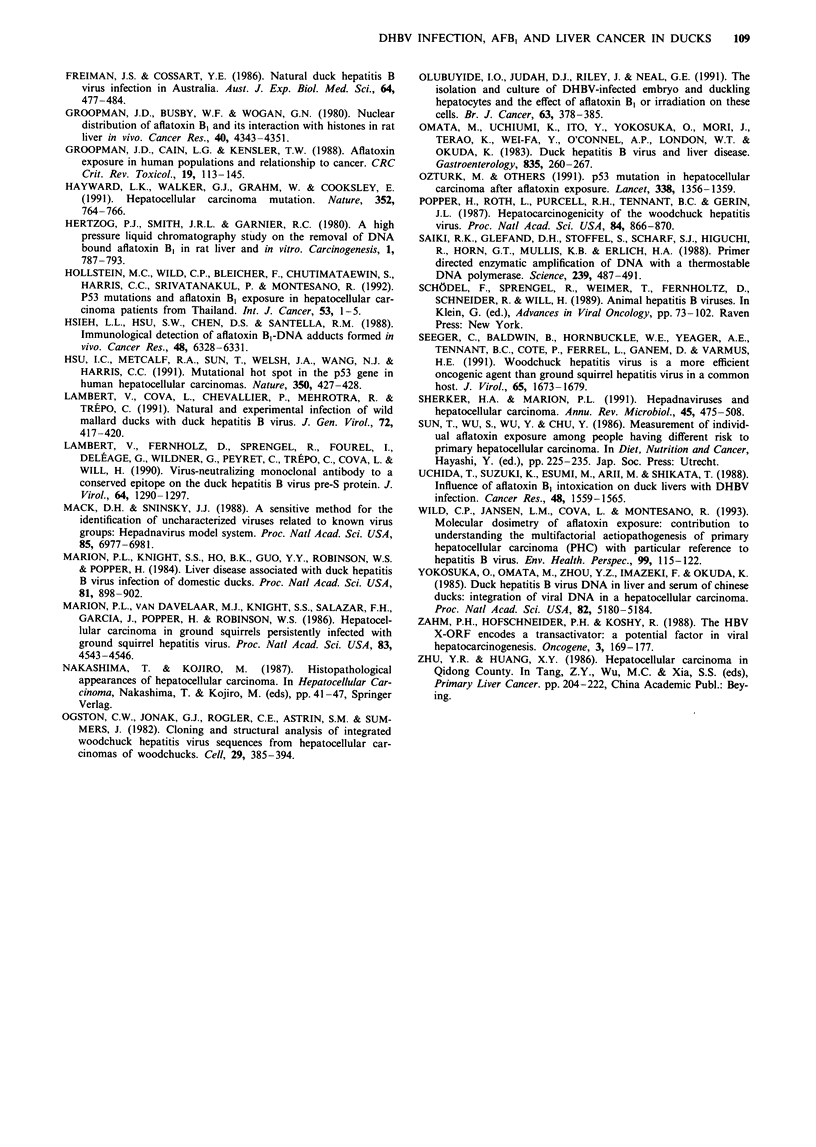# Duck hepatitis B virus infection, aflatoxin B1 and liver cancer in domestic Chinese ducks.

**DOI:** 10.1038/bjc.1994.16

**Published:** 1994-01

**Authors:** L. Cova, R. Mehrotra, C. P. Wild, S. Chutimataewin, S. F. Cao, A. Duflot, M. Prave, S. Z. Yu, R. Montesano, C. Trepo

**Affiliations:** INSERM U271, Lyon France.

## Abstract

**Images:**


Br. J. Cancer (1994), 69, 104–109 © Macmillan Press Ltd., 1994

### Duck hepatitis B virus infection, aflatoxin $B_1$ and liver cancer in domestic Chinese ducks

L. Cova[1], R. Mehrotra[2], C.P. Wild[3], S. Chutimataewin[3,5], S.F. Cao[4], A. Duflot[1], M. Prave[6], S.Z. Yu[4], R. Montesano[3] & C. Trepo[1]

[1]INSERM U271, 151 Cours A. Thomas, 69003 Lyon France, [2]KGMC, Lucknow, India, [3]International Agency for Research on Cancer, 150 Cours A. Thomas 69008 Lyon, France, [4]Shanghai Medical University, Shanghai, PRC, [5]Permanent address: National Cancer Institute, Bangkok, Thailand, [6]Ecole Nationale Veterinaire de Lyon, 69280 Marcy L'Etoile, France.

**Summary** The oncogenicity of Duck hepatitis B virus (DHBV) is unclear since hepatocellular carcinomas (HCCs) have been reported only in domestic ducks in Qidong, an area of China where hepatitis B virus (HBV) and aflatoxin $B_1$ ($AFB_1$) are risk factors for liver cancer in man. In order to better define the association between DHBV infection, $AFB_1$ and HCC we analysed a series of 16 duck liver samples collected from local farms in Qidong. HCC was found in eight and cirrhosis in one of these samples. Furthermore bile duct proliferation, characteristic of $AFB_1$ exposure in ducks and other animal species, was found in these ducks. Integration of DHBV DNA into cellular DNA was observed in only one out of four DHBV positive HCCs, indicating that viral integration is not prerequisite for tumour development. In four remaining HCCs the polymerase chain reaction (PCR) failed to show any DHBV DNA suggesting that liver tumours do occur in these ducks in the absence of DHBV infection. In addition, $AFB_1$-DNA adducts were detected by hplc-immunoassay in one such DHBV-negative tumour. In summary we demonstrate that risk factors other than DHBV, including $AFB_1$ exposure, may be important in duck liver carcinogenesis in Qidong.

All members of the hepadnavirus family, which includes hepatitis B viruses isolated from human (HBV), woodchucks (WHV), ground squirrels (GSHV) herons (HHV) and ducks (DHBV), share the ability to establish persistent infection in their hosts, are predominantly hepatotropic and have a relatively narrow host range, although they differ in their oncogenic potential (Schödel *et al.*, 1989). WHV seems more oncogenic than HBV and other known hepadnaviruses, since 100% of woodchucks experimentally infected with this virus develop hepatocellular carcinoma (HCC) within 17–36 months after infection (Popper *et al.*, 1987). In ground squirrels persistently infected with GSHV the latency is longer and the percentage of carriers developing HCC is lower (Marion *et al.*, 1986). This difference in oncogenicity seems to be related to the transforming capacity of these viruses since, despite similar levels of virus production and similar preneoplastic disease, WHV induces HCC more rapidly and more frequently than GSHV in woodchuck (Seeger *et al.*, 1991).

By contrast to mammalian hepadnaviruses only limited data is available on a possible association between DHBV infection and HCC. Pekin ducks congenitally infected with DHBV and followed for several years in various studies have not developed liver tumours (Freiman & Cossart 1986; Cova *et al.*, 1990; Cullen *et al.*, 1990; Lambert *et al.*, 1991). In fact HCC has been found only in domestic brown ducks from a single area of China, Qidong and only four Chinese duck HCCs had so far been described (Omata *et al.*, 1983; Marion *et al.*, 1984; Yokosuka *et al.*, 1985). Unlike the HCCs in human and woodchuck in which integrated HBV and WHV DNA in the host genome has regularly been observed (Brechot *et al.*, 1980, Ogston *et al.*, 1982), only a single case of Chinese duck HCC with integrated DHBV DNA has to date been reported (Yokosuka *et al.*, 1985).

Qidong is an area of high human HCC incidence in China, in which both HBV and aflatoxin $B_1$ ($AFB_1$) are risk factors (Sun *et al.*, 1986). Ducks are highly susceptible to the carcinogenic effects of $AFB_1$ and have been used in our laboratory (Cova *et al.*, 1990) and others (Uchida *et al.*, 1988; Cullen *et al.*, 1990) as an experimental system to study the role of hepadnavirus infection and $AFB_1$ exposure in the induction of liver tumours. The capacity of DHBV-infected duck hepatocytes to metabolise $AFB_1$ had been also investigated *in vitro* (Olubuyide *et al.*, 1991).

The high prevalence of human and duck liver cancer in Qidong may indicate the presence of common environmental risk factors. In the present study, in order to better define the association between DHBV infection, $AFB_1$ and HCC, we analysed a series of liver samples recently collected from domestic ducks in local farms in Qidong.

### Materials and methods

### *Liver samples*

Sixteen liver samples from adult (at least 3 year old) domestic Chinese brown ducks were collected from local farms in Qidong (1988–1989). These samples were not randomly collected, but selected for liver disease on routine pathologic examination of ducks. All ducks were raised on human domestic food which was predominantly corn. All samples were sent from China to France as frozen material. No sera were available.

### *Histological study of duck livers*

The frozen liver tissue was cut into small pieces, fixed in 10% buffered formalin, embedded in paraffin and sections were stained with standard histopathological techniques. The histological criteria for diagnosis of liver pathology were as follows; HCC were classified according to Nakashima and Kojiro (1987) as trabecular (sinusoidal), schirrous (sclerosing), pseudoglandular (acinar) and undifferentiated types. Portal inflammation was graded as absent, minor (+) involving few portal tracts and prominent (+ +) involving lymphocyte infiltrate which expanded the limiting plates and often bridged the adjacent portal tracts and was associated with hepatocyte necrosis. Focal parenchymal lymphocyte infiltrates were recorded. Biliary proliferation was graded as absent, mild (+), and prominent (+ +) the latter involving enlargement with extension into parenchyma along with portal tract enlargement. The absence of any feature suggestive of liver disease was classified as no pathology, but could include fatty change (steatosis) and focal hepatocyte necrosis.

Correspondence: L. Cova, INSERM U271, 151 Cours A. Thomas, 69003 Lyon France.
Received 28 April 1993; and in revised form 9 July 1993.

*Dot-blot and Southern blot analysis of liver DNA*

The liver tissue (0.2 g) was homogenised in liquid nitrogen, incubated with proteinase K (300 µg $ml^{-1}$) in the presence of 0.1% sodium dodecyl sulfate at 37°C for 3 h, thereafter proteins were removed by extraction with phenol/chloroform and DNA was precipitated with ethanol. Screening for the presence of DHBV DNA was performed by dot-blot hybridisation of 20 µg heat denatured liver DNA spotted in duplicate onto nitrocellulose (Schleicher & Schuell) and hybridised with radiolabelled DHBV probe as described (Cova *et al.*, 1990). For the Southern blot analysis, DNA samples (15 µg) were digested with restriction endonucleases (Boehringer Mannheim), subjected to electrophoresis through 0.8% (w/v) agarose (Sigma, USA) gel, transferred to nitrocellulose and hybridised to DHBV DNA radiolabelled by nick translation as described previously (Lambert *et al.*, 1990). The filters were washed, air dried and exposed at −70°C against Amersham hyperfilm MP as described (Cova *et al.*, 1990).

*Detection of DHBV by Polymerase Chain Reaction*

*Enzymatic amplification* DHBV-specific primers MD03 5'-CTCAAGCTTATCATCCATATA and MD33 5'-CTTGGA-TCCAATGGGCGTCGGTCT located in the most conserved region of the polymerase gene Mack & Sninsky (1988) were used. The position of these primers in the DHBV genome have been described by Mack & Sninsky (1988). Each reaction was performed essentially as described by Saiki *et al.* (1988) in a total volume of 50 µl containing the following: 10 mM Tris-HCl pH 8.4; 2 mM $MgCl_2$; 50 mM KCl; 0.01% gelatin, 200 µM of each dNTP, 1 µM each primer; 1 µg liver DNA; 0.5 units of Taq polymerase (Perkin-Elmer, Cetus USA). This mixture was overlayed with 100 µl of mineral oil and amplified for 35 cycles using a DNA thermal cycler (Perkin-Elmer, Cetus, USA). During each cycle, samples were heated to 94°C for 30 s, cooled to 55°C for 30 s, and incubated for 1 min at 72°C, with a final extension step of 10 min at 72°C.

*Analysis of amplified DNA* Ten µl of the PCR product were resolved in a 3% NiuSieve (FMC Corporation)-1% agarose (Sigma, USA) gel and transferred on a nylon membrane (Hybond $N^+$, Amersham) by alkaline blotting. Ten picomoles of the MD10 5'-CAGCCCTTTTCTCCTCCAT-CTCTTCACTACTGCCCTCGGA oligonucleotide probe, specific for the amplified DHBV fragment, were labelled by terminal transferase using ($\alpha-{}^{32}P$)dCTP (3000 m Ci $mmol^{-1}$, Amersham) as previously described (Baginski *et al.*, 1991). The filters were hybridised overnight at 42°C, thereafter excess probe was removed by several washes at 42°C as described (Chemin *et al.*, 1991). The filter was air-dried and then autoradiographed at −70°C using X-ray film (Hyperfilm MP; Amersham). The sensitivity of this PCR-Southern blot (PCR-SB) assay was estimated to 0.8 fg using serial dilutions of DNA from a DHBV- positive liver (data not shown). Each sample was tested at least three times in this SB-PCR.

*Analysis of aflatoxin DNA adduct*

Analysis was essentially performed as described previously (Hollstein *et al.*, 1992) with minor modification. DNA was extracted from frozen liver tissues (1.6–2.5 g) using phenol-chloroform and purified DNA was alkali treated to effect imidazole ring opening of the $AFB_1$-guanine adducts (8,9-dihydro-8-(2,6-diamino-4-oxo-3,4-dihydro-pyrimid-5-ylform-amido-)-9hydroxy) $AFB_1$ ($AFB_1$-Fapy). DNA was then acid hydrolysed (0.1 M HCl; 90°C, 20 min) to release $AFB_1$-imidazole ring opened guanine residues. The hydrolysed DNA was diluted to a final volume of 30 ml with phosphate buffered saline (pH 7.4) and loaded onto an activated Sep-pak C18 cartridge (Waters) for clean-up of $AFB_1$-Fapy residues. The $AFB_1$-Fapy containing eluate was subjected to hplc purification by reverse phase chromatography as described (Hollstein *et al.*, 1992) collecting 5 min fractions. Fractions were dried and the samples reconstituted in 250 µl PBS with 1% foetal calf serum for ELISA. A second series of injections using the same hplc system were made of aliquots of hydrolysed DNA kept prior to Sep-pak purification to determine the amount of DNA from adenine content. The ELISA was performed as described (Hollstein *et al.*, 1992; Chapot & Wild 1991). As the $AFB_1$-Fapy mainly eluted in two hplc fractions then around 600 fmoles is required in any sample for a positive result. The sensitivity of the assay is therefore defined for each sample as 600 fmoles divided by the quantity of DNA available.

### Results

*Occurrence and histological characteristics of liver tumours in Qidong ducks*

The liver pathology study indicates (Table I) that HCCs were found in eight (nos 31, 35, 37, 39, 40, 41, 42, 44) out of 16 ducks. The predominant morphological pattern of HCC was trabecular with tumour cells arranged in several thick cell plates and resembling hepatocytes with eosinophilic cytoplasm, prominent nucleoli and which were often separated by fibrous bands (Figure 1). The other morphological subtypes were; schirrous (nos 41, 42) and pseudoglandular (nos 35, 39, 40) (Table I). The schirrous type was characterised by the

**Figure 1** Hepatocellular carcinoma forming thick liver cell structures (duck no. 40). H&E × 250.

**Table I** DHBV infection and liver diseases in Qidong ducks

| | | | Associated histopathological features | |
|---|---|---|---|---|
| *Duck* | *DHBV*[d] | *HCC* | *Lymphocyte infiltration* | *Biliary proliferation* |
| 30 | − | − | + | − |
| 31 | − | + | + + | + |
| 32 | − | − | + | − |
| 33 | + | − | + + | − |
| 34* | + | − | + + | + + |
| 35 | − | +[b] | + + | + |
| 36 | − | − | + | + |
| 37 | + | +[c] | − | − |
| 38 | + | − | + | − |
| 39 | − | +[b] | + | − |
| 40 | − | +[b,c] | − | − |
| 41 | + | +[a,c] | − | − |
| 42 | + | +[a] | − | − |
| 43 | + | − | − | − |
| 44 | + | + | − | − |
| 45 | − | − | − | − |

[a]Were scirrhous type HCC, [b]pseudoglandular, [c]undifferentiated type. *Duck no. 34 had liver cirrhosis. [d]Screening of liver samples for the presence of DHBV DNA was performed by dot-blot hybridisation of heat-denatured liver DNA (see Materials and methods).

presence of thick bands of fibrous septa, clearly seen at high magnification (Figure 2), which had isolated groups of tumour cells resembling hepatocytes. The pseudoglandular type HCC showed a variety of gland like cystic spaces formed by central degeneration or breakdown of otherwise solid trabeculae. In some ducks (nos 37, 40, 41) the tumour cells had a tendency towards poor differentiation along with the presence of multinucleated or single nucleus tumour giant cells and erythropoesis foci mostly composed of pleomorphic clumps of erytheroid and myeloid precursors together with megakaryocytes. Liver cirrhosis (no 34) was characterised by well formed regenerative hyperplastic nodules separated by fibrous septa containing periportal periseptal lymphocyte infiltration (+ +), biliary proliferation (+ +), and occasional hepatocyte necrosis. The other non neoplastic lesions were lymphocyte infiltrate (+/+ +) present in eight and biliary proliferation (Figure 3) present in four ducks. In addition, foci of hyperplastic eosinophilic hepatocytes composed of large cells with deeply stained cytoplasm were present. No parasites or ground glass hepatocytes were present.

### *Detection of DHBV DNA in duck livers*

DHBV DNA was initially detected by a conventional blot hybridisation test in eight out of 16 (50%) liver samples (Table I). There were four non tumorous livers (nos 33, 34, 38, 43) and four HCCs (nos 37, 41, 42, 44) which were DHBV positive (Table I). However, in the remaining four HCCs (nos 31, 35, 39, 40) DHBV DNA was undetectable by dot-blot hybridisation (Table I). PCR was performed using DHBV specific primers to assess whether the failure of DHBV DNA detection in these four HCCs was related to the absence of virus in these livers or to the low level of viral replication undetectable by dot-blot hybridisation. Agarose gel analysis of amplification products revealed the presence of the predicted 124 bp band in samples of liver DNA derived from two French ducks (Figure 4a lanes 1 and 8), known to be congenitally infected with DHBV (Cova *et al.*, 1990), as well as the Qidong duck no 42 (Figure 4a, lane 6) shown by dot-blot to be DHBV-positive (Table I). These positive results were confirmed by SB-hybridisation of the gel with the DHBV-specific MD 10 oligonucleotide probe (Figure 4b). None of the four Qidong duck HCCs (Figure 4a, b, lanes 2–5) which were negative for DHBV by dot blot or a control liver sample from an uninfected French duck (lane 7) gave a hybridisation signal with MD 10 probe. Taken together these results suggest that liver cancer in Qidong ducks occur in the absence of DHBV-DNA detectable by SB-PCR (detection limit: 0.8 fg of DHBV DNA per μg of liver DNA).

### *State of viral DNA in HCCs*

Integration of viral DNA sequences into cellular DNA is characteristic of hepadnavirus-induced HCC. To determine whether integration of DHBV DNA occurs in the liver tumours of Qidong ducks, we examined DNA from virus-positive HCC nos 37, 41, 42, 44. The analysis by Southern blot of undigested liver DNA revealed the presence of a high molecular weight band in only one (no 42) out of the four DHBV positive HCCs (data not shown). The DNA prepared from this tumour was further analysed by digestion with restriction endonucleases (KpnI, XhoI, EcoRI), each having a single recognition site in this DHBV isolate (Figure 5a, b). The high molecular weight band observed in uncut DNA from this HCC was converted after digestion with KpnI, XhoI or EcoRI to several slowly migrating DNA fragments of 5 to 11 kb size (Figure 5b), indicating that DHBV

**Figure 2** Hepatocellular carcinoma schirrous type. Small groups of tumour cells are separated by fibrous tissue (duck no. 41) H&E × 150.

**Figure 3** Biliary proliferation present in fibrous septa of a cirrhotic nodule (duck no. 34) H&E × 150.

**Figure 4** PCR amplification of DHBV in the duck livers. **a**, Ethidium bromide-stained agarose gel. **b**, Autoradiograph of gel in **a**, Southern blotted and hybridised with DHBV specific probe MD 10. Lanes 1 and 8 liver DNA of French ducks nos 562 and 447 congenitally infected with DHBV (positive controls); lanes 2 to 6 respectively, DNAs from Qidong duck HCCs nos 31, 35, 39, 40, 42; lane 7 DNA from an uninfected French duck no 256 (negative control). Arrow indicates 124 base pairs. Lane M, Hae III-cleaved pBR322 DNA served as a molecular weight marker.

**Figure 5** Southern blot analysis of DHBV DNAs obtained from Qidong ducks **a**, Liver from duck 33 which had no hepatic neoplasma. **b**, HCC from duck 42. Purified DNA was undigested (lane 1), digested with KpnI (lane 2), XhoI (lane 3), EcoRI (lane 4). The size markers (in kilobases) are HindIII-digested lambda DNA.

sequences were integrated into cellular DNA. As expected, such fragments were not observed in the DNA prepared from the non neoplastic tissue (Figure 5a).

### *$AFB_1$ adducts detection*

DNA was extracted from eight liver samples, including seven HCCs from Qidong. Of these HCCs the non neoplastic tissue was available from only one sample, duck no. 31. The other samples were from the tumorous part of the liver. $AFB_1$-N7-guanine is the adduct initially formed in DNA after $AFB_1$ exposure but is either rapidly lost due to depurination (half life; eight to 12 h) (Groopman *et al.*, 1980) followed by excretion in the urine or is converted to $AFB_1$-Fapy which is more stable and persistent. The latter adduct is the major one in the rat 48 h after treatment (Hertzog *et al.*, 1980) and has been detected in duck liver after injection with ($^3H$) $AFB_1$ (Cova *et al.*, 1990). In the present study DNA was alkali treated prior to hydrolysis to convert any $AFB_1$-N7-guanine to $AFB_1$-Fapy. The presence of $AFB_1$-Fapy was detected in one of the liver samples (no 31) (Figure 6a). The basis of this identification is (i) the inhibition in immmunoassay using an aflatoxin-specific antibody and (ii) the co-chromatography of this inhibitory material with authentic $AFB_1$-Fapy adduct (Figure 6b). Two separate analyses of this liver were made starting each time from a different piece of liver tissue and both analyses were positive, the level of adducts determined being 4.61 and 1.73 ng $AFB_1$-Fapy per mg DNA (mean = 3.17 ng mg$^{-1}$ or 6.38 pmoles per mg DNA). The quantitative differences in the two results could reflect differences in localisation of adducts within the liver and/or interassay variation. It is perhaps significant that sample 31 was the only HCC sample for which non neoplastic tissue was available. An attempt was made to measure the $AFB_1$-Fapy in the tumour part of the liver from the same duck but only 0.15 mg DNA was available giving a detection limit of around three pmoles $AFB_1$-Fapy per mg DNA and the sample was below this limit of detection.

### Discussion

There are limited data on the correlation between DHBV infection and liver disease occurring in domestic ducks from

**Figure 6** $AFB_1$-Fapy adducts in duck liver DNA. $AFB_1$-Fapy adducts were assayed as described in Materials and methods. **a**, Shows the inhibition in ELISA by each hplc fraction for duck 31 (●) and a control duck from an area where dietary $AFB_1$ exposure would be expected to be low, (○). **b**, Shows the same profile for $AFB_1$-Fapy standard injected onto the hplc under the same conditions (▲).

Qidong county since for previous studies (Marion *et al.*, 1984; Omata *et al.*, 1983) only a small number of liver tumours was available most of them being paraffin embedded material which considerably increased the difficulty in their molecular analysis. We report here our investigations on liver cancer, DHBV infection, viral DNA integration and $AFB_1$ adducts in a panel of frozen liver samples from Qidong ducks.

In previous studies only four HCC's were described and all of them were well differentiated HCC of trabecular type (Omata *et al.*, 1983; Marion *et al.*, 1984; Yokosuka *et al.*, 1985). In the present study of a larger panel of eight HCCs we have observed a range of different morphological types e.g. schirrous, pseudoglandular and even undifferentiated HCC. The presence of liver cirrhosis was observed by us and by Omata *et al.* (1983) in Qidong ducks. Another interesting pathological feature of our study, not previously reported, was the biliary proliferation both in ducks with and without HCC from the Qidong area. Biliary proliferation has not been reported to be associated with DHBV infection but is seen in ducks experimentally exposed to $AFB_1$ (Uchida *et al.*, 1988; Cova *et al.*, 1990; Cullen *et al.*, 1990). While our observations of biliary proliferation are therefore consistent with $AFB_1$ exposure we cannot rule out that it could have been a result of exposures to factors other than aflatoxin.

In two previous studies liver disease and HCC in ducks from Qidong were not always associated with detectable virus (Marion *et al.*, 1984; Omata *et al.*, 1983). It has been suggested that a low level of DHBV replication might occur in some such livers, although it was at the limit of sensitivity of a dot blot assay. We have taken advantage of the high specificity and sensitivity of SB-PCR, which has been found $10^4$ times more sensitive than the dot-blot assay (Chemin et al., 1991), to search for the presence of DHBV DNA in the Chinese duck HCCs. We demonstrated here that in four out of eight HCCs SB-PCR failed to show any DHBV DNA, suggesting that liver tumours occur in the ducks from Qidong in the absence of DHBV infection. The absence of detectable viral DNA is not due to failure of DHBV specific primers to anneal to the DNA of the Chinese DHBV isolate since the primer set chosen for this study, located in the highly conserved region of hepadnavirus DNA polymerase, permitted the amplification of DHBV DNA in infected livers originating from both French and Qidong ducks.

The association between HBV, WHV and GSHV DNA integration and HCC has been demonstrated as frequently associated with malignant transformation (Brechot et al., 1980; Ogston et al., 1982; Marion et al., 1986). The previous reports on Chinese duck HCC revealed only a single case of DHBV DNA integration (Yokosuka et al., 1985) and in this study we report a second case. The integration of DHBV DNA is not a prerequisite for HCC development since it was observed in only one out of four DHBV-positive HCCs analysed in the present study. The results of Cullen et al. (1990) suggested a possible role of $AFB_1$ in the integration of DHBV into high molecular weight DNA. The significance of DHBV DNA integration observed in Qidong ducks remains to be clarified. It is of interest to note that the integrated DHBV DNA observed by us in duck no 42 was associated with an intense ongoing viral DNA replication. This is similar to the woodchuck HCC, but different to most HBV-associated human HCC in which replication of virions is diminished or even absent (Sherker & Marion, 1991).

The exposure of domestic ducks in Qidong to $AFB_1$ has been suggested, but never demonstrated. However, given the high aflatoxin content of maize in Qidong (Zhu & Huang, 1986) the exposure of both humans and domestic animals would be expected (Sun et al., 1986). In the present study one duck (no 31) was positive for the presence of $AFB_1$-DNA adducts in liver. This duck had no DHBV infection and had significant biliary proliferation, a feature of aflatoxin exposure in ducks. As mentioned above, this was the only sample where non neoplastic tissue was available. The lack of detectable $AFB_1$-DNA adducts in the other ducks could therefore have been a result of only tumour tissue being available for analysis or alternatively, the $AFB_1$ exposure could have been lower in these ducks. The levels of $AFB_1$-Fapy have previously been measured after a single dose of $AFB_1$ in adult ducks (Cova et al., 1990) and ducklings (Wild et al., 1993). A single dose of 20 $\mu$g $AFB_1$/kg in adult ducks gave a mean $0.57 \pm 0.12$ ng $AFB_1$-Fapy $mg^{-1}$ DNA and a single dose of 2 $\mu g\ kg^{-1}$ in ducklings gave $0.025 \pm 0.002$ ng $mg^{-1}$. The present level is therefore about five times higher than a single dose of 20 $\mu g\ kg^{-1}$. $AFB_1$-Fapy can accumulate upon repeated exposures in rats (Croy & Wogan, 1981) and persist up to 19 weeks post treatment (Groopman et al., 1988) therefore the chronic dose producing this level of DNA adduct could be much lower than 20 $\mu g\ kg^{-1}$. The duck adduct level is also similar to those reported in human liver samples from hepatocellular carcinoma patients in Taiwan (Hsieh et al., 1988) and experimentally in rats and trout in which HCC was induced by $AFB_1$ (Bechtel, 1989).

As only one duck was positive for $AFB_1$-DNA adducts in this study further investigation of food contamination and aflatoxin-DNA adducts in duck liver are required in order to confirm the suggested importance of this carcinogen in liver cancer development in Qidong ducks. Since Qidong is the only area where liver cancer has been reported in ducks the possibly important role of $AFB_1$ raises the question of whether DHBV is indeed an oncogenic virus or not. The differences in the oncogenicity between mammalian and avian hepadnaviruses might be related not only to the milder liver disease induced by DHBV in its host, but also to a direct effect of viral gene products such as the X gene which can transactivate cellular transforming genes (Zahm et al., 1988), but is lacking in DHBV.

Recently, a high frequency of a mutational hotspot in codon 249 of the p53 tumour suppressor gene was found in human hepatomas from patients in Qidong and Southern Africa but not in the HCCs from several geographic locations in which $AFB_1$ is not a risk factor (Bressac et al., 1991; Hsu et al., 1991; Ozturk et al., 1991) supporting the important role of $AFB_1$ in liver carcinogenesis in some geographical areas. This specific p53 mutation may be independent of HBV infection (Hayward et al., 1991), although the different mechanisms of a possible interaction between HBV infection and exposures to $AFB_1$ in liver carcinogenesis are important to define and have been recently discussed (Wild et al., 1993). The ongoing search for the p53 gene mutation in Qidong duck hepatomas will be informative in this respect.

We thank G. Mollon for excellent photography. This work was supported in part by grant 6735 from Association pour la Recherche contre le Cancer (ARC) and by grant 104-1 from Indo-French Centre for the Promotion of Advanced Research (IFCPAR). A Duflot is the recipient of a fellowship from Ligue Nationale contre le Cancer.

Abbreviations: DHBV, Duck hepatitis B virus; HCC hepatocellular carcinoma; $AFB_1$ aflatoxin $B_1$.

### References

BAGINSKI, I., CHEMIN, I., BOUFFARD, P., HANTZ, O. & TREPO, C. (1991). Detection of polyadenylated RNA in hepatitis B virus-infected peripheral blood mononuclear cells by polymerase chain reaction. *J. Inf. Dis.*, **163**, 996–1000.

BECHTEL, D.H. (1989). Molecular dosimetry of hepatic aflatoxin $B_1$-DNA adducts: linear correlation with hepatic cancer risk. *Reg. Toxicol. Pharm.*, **10**, 74–81.

BRECHOT, C., POURCEL, C., LOISE, A., RAIN, B. & TIOLLAIS, P. (1980). Presence of integrated hepatitis B virus DNA sequences in cellular DNA of human hepatocellular carcinoma. *Nature*, **286**, 533–535.

BRESSAC, B., KEW, M., WANDS, J. & OZTURK, M. (1991). Selective G to T mutation of p53 gene in hepatocellular carcinoma from southern Africa. *Nature*, **350**, 429–431.

CHAPOT, B. & WILD, C.P. (1991). ELISA for quantitation of aflatoxin-albumin adducts and their application to human exposure assessment In *Techniques in Diagnostic Pathology*, Warhol, M.V., Velzen, D. & Bullock, G.R. (eds), Vol 2, pp 135–155, Academic Press.

CHEMIN, I., BAGINSKI, I., PETIT, M.A., ZOULIM, F., PICHOUD, C., CAPEL, F., HANTZ, O. & TREPO, C. (1991). Correlation between HBV DNA detection by polymerase chain reaction and pre-S1 antigenemia in symptomatic and asymptomatic hepatitis B virus infections. *J. Med. Virol.*, **33**, 51–57.

COVA, L., WILD, C.P., MEHROTRA, R., TURUSOV, V., SHIRAI, T., LAMBERT, V., JACQUET, C., TOMATIS, L., TRÉPO, C. & MONTESANO, R. (1990). Contribution of aflatoxin $B_1$ and hepatitis B virus infection in the induction of liver tumors in ducks. *Cancer Res.*, **50**, 2156–2163.

CROY, R.G. & WOGAN, G.N. (1981). Temporal pattern of covalent DNA adducts in rat liver after single and multiple doses of aflatoxin $B_1$. *Cancer Res.*, **41**, 197–203.

CULLEN, J.M., MARION, P.L., SHERMAN, G.J., HONG, X. & NEWBOLD, J.E. (1990). Hepatic neoplasms in aflatoxin $B_1$-treated, congenital duck hepatitis B virus-infected and virus-free Pekin ducks. *Cancer Res.*, **50**, 4072–4080.

FREIMAN, J.S. & COSSART, Y.E. (1986). Natural duck hepatitis B virus infection in Australia. *Aust. J. Exp. Biol. Med. Sci.*, **64**, 477–484.

GROOPMAN, J.D., BUSBY, W.F. & WOGAN, G.N. (1980). Nuclear distribution of aflatoxin B$_1$ and its interaction with histones in rat liver *in vivo*. *Cancer Res.*, **40**, 4343–4351.

GROOPMAN, J.D., CAIN, L.G. & KENSLER, T.W. (1988). Aflatoxin exposure in human populations and relationship to cancer. *CRC Crit. Rev. Toxicol.*, **19**, 113–145.

HAYWARD, L.K., WALKER, G.J., GRAHM, W. & COOKSLEY, E. (1991). Hepatocellular carcinoma mutation. *Nature*, **352**, 764–766.

HERTZOG, P.J., SMITH, J.R.L. & GARNIER, R.C. (1980). A high pressure liquid chromatography study on the removal of DNA bound aflatoxin B$_1$ in rat liver and *in vitro*. *Carcinogenesis*, **1**, 787–793.

HOLLSTEIN, M.C., WILD, C.P., BLEICHER, F., CHUTIMATAEWIN, S., HARRIS, C.C., SRIVATANAKUL, P. & MONTESANO, R. (1992). P53 mutations and aflatoxin B$_1$ exposure in hepatocellular carcinoma patients from Thailand. *Int. J. Cancer*, **53**, 1–5.

HSIEH, L.L., HSU, S.W., CHEN, D.S. & SANTELLA, R.M. (1988). Immunological detection of aflatoxin B$_1$-DNA adducts formed *in vivo*. *Cancer Res.*, **48**, 6328–6331.

HSU, I.C., METCALF, R.A., SUN, T., WELSH, J.A., WANG, N.J. & HARRIS, C.C. (1991). Mutational hot spot in the p53 gene in human hepatocellular carcinomas. *Nature*, **350**, 427–428.

LAMBERT, V., COVA, L., CHEVALLIER, P., MEHROTRA, R. & TRÉPO, C. (1991). Natural and experimental infection of wild mallard ducks with duck hepatitis B virus. *J. Gen. Virol.*, **72**, 417–420.

LAMBERT, V., FERNHOLZ, D., SPRENGEL, R., FOUREL, I., DELÉAGE, G., WILDNER, G., PEYRET, C., TRÉPO, C., COVA, L. & WILL, H. (1990). Virus-neutralizing monoclonal antibody to a conserved epitope on the duck hepatitis B virus pre-S protein. *J. Virol.*, **64**, 1290–1297.

MACK, D.H. & SNINSKY, J.J. (1988). A sensitive method for the identification of uncharacterized viruses related to known virus groups: Hepadnavirus model system. *Proc. Natl Acad. Sci. USA*, **85**, 6977–6981.

MARION, P.L., KNIGHT, S.S., HO, B.K., GUO, Y.Y., ROBINSON, W.S. & POPPER, H. (1984). Liver disease associated with duck hepatitis B virus infection of domestic ducks. *Proc. Natl Acad. Sci. USA*, **81**, 898–902.

MARION, P.L., VAN DAVELAAR, M.J., KNIGHT, S.S., SALAZAR, F.H., GARCIA, J., POPPER, H. & ROBINSON, W.S. (1986). Hepatocellular carcinoma in ground squirrels persistently infected with ground squirrel hepatitis virus. *Proc. Natl Acad. Sci. USA*, **83**, 4543–4546.

NAKASHIMA, T. & KOJIRO, M. (1987). Histopathological appearances of hepatocellular carcinoma. In *Hepatocellular Carcinoma*, Nakashima, T. & Kojiro, M. (eds), pp. 41–47, Springer Verlag.

OGSTON, C.W., JONAK, G.J., ROGLER, C.E., ASTRIN, S.M. & SUMMERS, J. (1982). Cloning and structural analysis of integrated woodchuck hepatitis virus sequences from hepatocellular carcinomas of woodchucks. *Cell*, **29**, 385–394.

OLUBUYIDE, I.O., JUDAH, D.J., RILEY, J. & NEAL, G.E. (1991). The isolation and culture of DHBV-infected embryo and duckling hepatocytes and the effect of aflatoxin B$_1$ or irradiation on these cells. *Br. J. Cancer*, **63**, 378–385.

OMATA, M., UCHIUMI, K., ITO, Y., YOKOSUKA, O., MORI, J., TERAO, K., WEI-FA, Y., O'CONNEL, A.P., LONDON, W.T. & OKUDA, K. (1983). Duck hepatitis B virus and liver disease. *Gastroenterology*, **835**, 260–267.

OZTURK, M. & OTHERS (1991). p53 mutation in hepatocellular carcinoma after aflatoxin exposure. *Lancet*, **338**, 1356–1359.

POPPER, H., ROTH, L., PURCELL, R.H., TENNANT, B.C. & GERIN, J.L. (1987). Hepatocarcinogenicity of the woodchuck hepatitis virus. *Proc. Natl Acad. Sci. USA*, **84**, 866–870.

SAIKI, R.K., GLEFAND, D.H., STOFFEL, S., SCHARF, S.J., HIGUCHI, R., HORN, G.T., MULLIS, K.B. & ERLICH, H.A. (1988). Primer directed enzymatic amplification of DNA with a thermostable DNA polymerase. *Science*, **239**, 487–491.

SCHÖDEL, F., SPRENGEL, R., WEIMER, T., FERNHOLTZ, D., SCHNEIDER, R. & WILL, H. (1989). Animal hepatitis B viruses. In Klein, G. (ed.), *Advances in Viral Oncology*, pp. 73–102. Raven Press: New York.

SEEGER, C., BALDWIN, B., HORNBUCKLE, W.E., YEAGER, A.E., TENNANT, B.C., COTE, P., FERREL, L., GANEM, D. & VARMUS, H.E. (1991). Woodchuck hepatitis virus is a more efficient oncogenic agent than ground squirrel hepatitis virus in a common host. *J. Virol.*, **65**, 1673–1679.

SHERKER, H.A. & MARION, P.L. (1991). Hepadnaviruses and hepatocellular carcinoma. *Annu. Rev. Microbiol.*, **45**, 475–508.

SUN, T., WU, S., WU, Y. & CHU, Y. (1986). Measurement of individual aflatoxin exposure among people having different risk to primary hepatocellular carcinoma. In *Diet, Nutrition and Cancer*, Hayashi, Y. (ed.), pp. 225–235. Jap. Soc. Press: Utrecht.

UCHIDA, T., SUZUKI, K., ESUMI, M., ARII, M. & SHIKATA, T. (1988). Influence of aflatoxin B$_1$ intoxication on duck livers with DHBV infection. *Cancer Res.*, **48**, 1559–1565.

WILD, C.P., JANSEN, L.M., COVA, L. & MONTESANO, R. (1993). Molecular dosimetry of aflatoxin exposure: contribution to understanding the multifactorial aetiopathogenesis of primary hepatocellular carcinoma (PHC) with particular reference to hepatitis B virus. *Env. Health. Perspec.*, **99**, 115–122.

YOKOSUKA, O., OMATA, M., ZHOU, Y.Z., IMAZEKI, F. & OKUDA, K. (1985). Duck hepatitis B virus DNA in liver and serum of chinese ducks: integration of viral DNA in a hepatocellular carcinoma. *Proc. Natl Acad. Sci. USA*, **82**, 5180–5184.

ZAHM, P.H., HOFSCHNEIDER, P.H. & KOSHY, R. (1988). The HBV X-ORF encodes a transactivator: a potential factor in viral hepatocarcinogenesis. *Oncogene*, **3**, 169–177.

ZHU, Y.R. & HUANG, X.Y. (1986). Hepatocellular carcinoma in Qidong County. In Tang, Z.Y., Wu, M.C. & Xia, S.S. (eds), *Primary Liver Cancer*. pp. 204–222, China Academic Publ.: Beying.